# Supplementary material for: Full polarization control of photons with evanescent wave coupling in the ultra subwavelength gap of photonic molecules
Source: Light Sci Appl. 2025 Mar 5;14:114. doi: 10.1038/s41377-025-01794-1 (PMC11882835; doi:10.1038/s41377-025-01794-1)
Supplement: Supplementary file 1 — Supplementary Information for Full Polarization Control of Photons with Evanescent Wave Coupling in the Ultra-Subwavelength Gap of Photonic Molecules [file 41377_2025_1794_MOESM1_ESM.pdf]

Supplementary Information for Full Polarization Control of Photons with Evanescent Wave Coupling in the Ultra Subwavelength Gap of Photonic Molecules

Rui Zhu,[1,2,*] Chenjiang Qian,[1,2,*] Shan Xiao,[1,2,*] Jingnan Yang,[3] Sai Yan,[1,2] Hanqing Liu,[4] Deyan Dai,[4] Hancong Li,[3] Longlong Yang,[3] Xiqing Chen,[3] Yu Yuan,[1,2] Danjie Dai,[1,2] Zhanchun Zuo,[1,2,†] Haiqiao Ni,[4] Zhichuan Niu,[4] Can Wang,[1,2,‡] Kuijuan Jin,[1,2] Qihuang Gong,[3] and Xiulai Xu[3,5,6,§]

[1]*Beijing National Laboratory for Condensed Matter Physics, Institute of Physics, Chinese Academy of Sciences, Beijing 100190, China*

[2]*School of Physical Sciences, University of Chinese Academy of Sciences, Beijing 100049, China*

[3]*State Key Laboratory for Mesoscopic Physics and Frontiers Science Center for Nano-optoelectronics, School of Physics, Peking University, 100871 Beijing, China*

[4]*State Key Laboratory of Superlattices and Microstructures, Institute of Semiconductors Chinese Academy of Sciences, Beijing 100083, China*

[5]*Peking University Yangtze Delta Institute of Optoelectronics, Nantong, Jiangsu 226010, China*

[6]*Collaborative Innovation Center of Extreme Optics, Shanxi University, Taiyuan, Shanxi 030006, China*

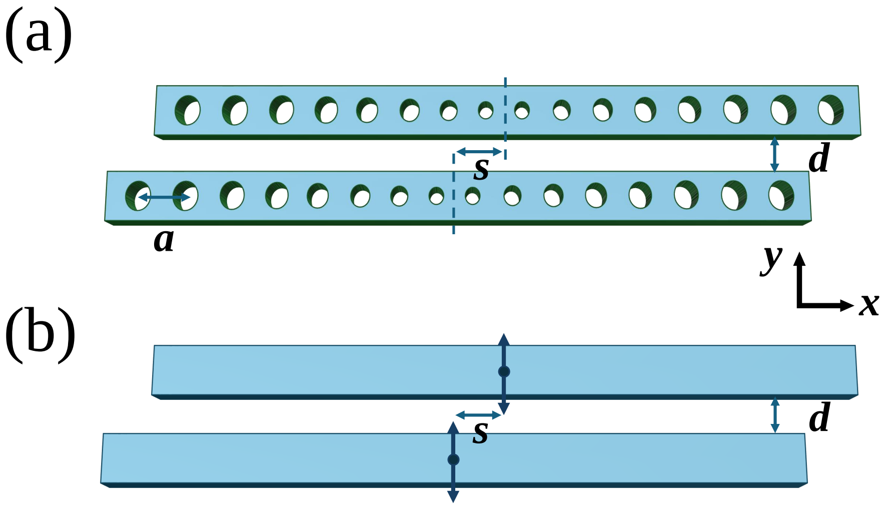

FIG. S1. (a) Model of the photonic molecule with two identical nanobeam cavities, used in the calculation of supermodes and in the pattern profile for nanofabrication. (b) Qualitative model of the nanobeam with a y polarized dipole as an approximation to the nanobeam cavity, used in the calculation of far-field polarization properties in Fig. 5 in the main text.

I. METHODS IN THEORETICAL CALCULATIONS

In this work, we use the three-dimensional (3D) finite element method (FEM) and finite-difference time-domain (FDTD) method to calculate the supermodes of photonic molecules (PMs). Fig. S1 (a) schematically depicts how we design the nanobeam cavity with a photonic crystal array of nanoholes, which was used for the FEM calculation presented in Figs. $1-3$ in the main text. A single nanobeam cavity consists of 32 nanoholes. At each end, ten identical and periodic nanoholes with a lattice constant $a = 260$ nm and a radius $r = 68.8$ nm functioning as mirrors to reflect the photons (for brevity, only two are drawn in the schematic at each end in Fig. S1(a)). The twelve nanoholes at the center (six on each side) have a linearly changing a from 260 to 200 nm and r from 68.8 to 40.3 nm for the nanoholes from the sides to the center. Because the evanescent wave coupling in the ultra subwavelength gap is the key to a polarization control, we use a minimum mesh of 2 nm for the gap between the two nanobeam cavities. A perfect match layer (PML) is set at the outermost region. In the calculations, the air gap d changes from 0 to 400 nm with an interval of 5 nm for each step, while the lateral shift s changes from 0 to 650 nm with an

* These authors contributed equally to this work.
† zczuo@iphy.ac.cn
‡ canwang@iphy.ac.cn
§ xlxu@pku.edu.cn

interval of 13 nm. The lateral displacement s results in an increase in the lengths of the two nanobeams. Due to the consistency between the model in Fig. S1 (a) and the fabricated PMs in the experiment, the calculation results in Fig. 1 and 2 exhibit good agreement with the measurements. The model in Fig. S1(a) is also used for the calculation of far-field polarization. We optimize the simulation area and mesh settings of the model using the FDTD method, and calculate the polarization properties as the function of gap and shift. The calculation results are presented in Fig. 4 and well reproduce the experimental observations.

The calculation of far-field polarization using the model in Fig. S1(a) with the small mesh size takes a very long time, and thereby, the calculation with the varying of both gap and shift is not feasible. As such, we use a simple qualitative model as shown in Fig. S1(b) for the calculation of far-field polarization using the FDTD method, corresponding to Fig. 5 in the main text. The width and depth of the long nanobeam are both 180 nm. The mesh in the FDTD simulation is set to 10 nm. We use the y polarized dipole source with an emission wavelength of 1080 nm to simulate the y polarized mode of a single cavity. For the PM, the two dipoles have the same phase for the approximation of the S mode and the opposite phase for the AS mode. As discussed in the main text, although this qualitative model is not entirely the same as the PM in Fig. S1(a), the essential physics that the domination of the evanescent field near the nanobeam is well reproduced in the qualitative model. This crucial feature gives the key results, i.e., the change of regime from far-field wave coupling to evanescent wave coupling when the gap is below the threshold polarization results, which is qualitatively consistent with experimental results.

II. METHODS IN EXPERIMENT

Based on optimized parameters through numerical simulations, we fabricated and measured nanobeam PMs with different d and s. As shown in Fig. S2(a), The samples in this work were grown by molecular-beam epitaxy and made up of three layers including a 150-nm-thickness GaAs layer, a 1-μm-thickness AlGaAs sacrifice layer, and a GaAs substrate. One layer of InGaAs quantum dots (QDs) was embedded in the middle of the GaAs layer as excitation sources for cavity modes. The PL spectral range of the QD ensemble is about 950 nm to 1100 nm. The fabrication procedures of the PMs are presented in Fig. S2(b). Elec-

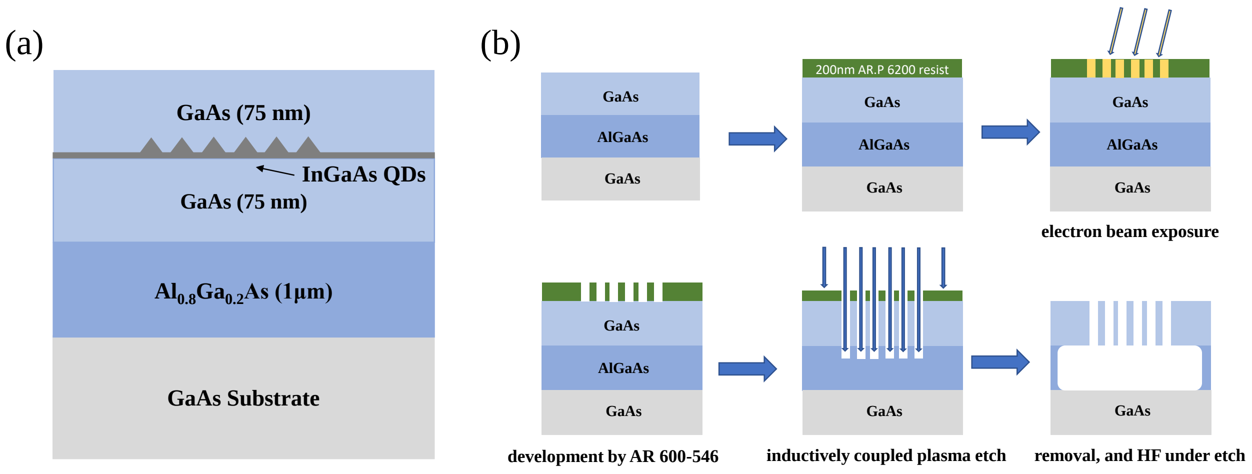

FIG. S2. (a) The structure of the sample grown by molecular beam epitaxy. InGaAs QDs are embedded in the GaAs layer. The AlGaAs sacrificial layer between the top GaAs layer and the bottom GaAs substrate has a thickness of 1 μm. (b) Fabrication procedures of the PM, mainly include electron beam lithography, inductively coupled plasma etching, and HF wet under etching.

tron beam lithography was used to pattern the PM structures with a mask on the sample. After development, inductively coupled plasma was used to etch unprotected GaAs regions by the mask until the AlGaAs layer was exposed with patterns on the mask transferred onto the GaAs layer. In the end, wet etching with HF solutions was used to remove the AlGaAs sacrificial layer and form suspended PM structures. Different parameters are used to fabricate the devices, and the accurate parameters of each device after fabrication are characterized using scanning electron microscopy.

The PM devices were mounted in a cryostat cooled down by liquid helium flow to achieve a low temperature of about 4.2 K. InGaAs QDs were excited by a cw laser with a wavelength of 532 nm, and then the PL emission of ensemble QDs further excited the cavity modes resulting in sharp peaks in the spectra. The PL spectra were collected by a high NA (0.82) objective and then analyzed by a spectrometer with InGaAs detectors. The polarization resolved spectra were collected by filtering the PL signals through a rotating half-wave plate and a polarizer.

III. RESULTS IN CONTROL CASES

The single dipole model in Fig. S1(b) well describes the full polarization control and reveals the key role of evanescent wave coupling. One might wonder that the electric field of

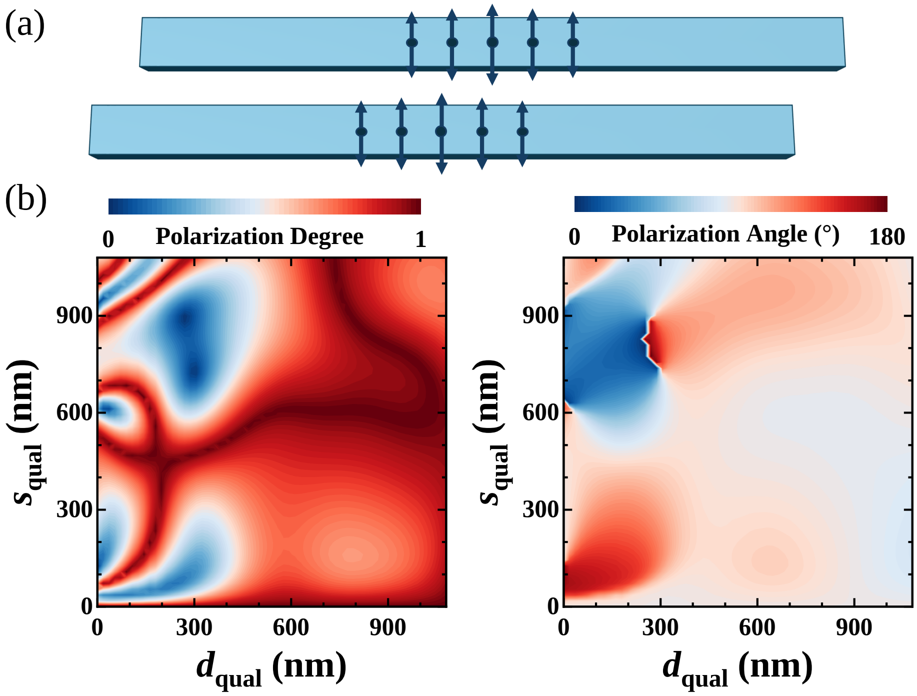

FIG. S3. (a) The qualitative model in Fig. S1(b) has multiple dipoles in one nanobeam. (b) Calculation results with polarization degree and angle as functions of gap d_{qual} and shift s_{qual} for the model in (a).

the real nanobeam cavity as shown in Fig. 1 has multiple antinodes, whilst the qualitative model in Fig. S1(b) only has one antinode at the position of the dipole. As such, we use the model with multiple dipoles for a better approximation of the real nanobeam cavity, as shown in Fig. S3(a). The amplitude of the first and second dipole away from the center is 0.9 and 0.7 times the amplitude of the dipole at the center, respectively, to simulate the Gaussian envelope of the electric field in the real nanobeam cavity. Calculation results are presented in Fig. S3(b). As shown, the multi-dipole model generally gives the same results compared to the one-dipole model shown in Fig. 5 of the main text.

To further strengthen the key role of evanescent wave coupling in full polarization control, we additionally calculate with the model in Fig. S1(b) but with the nanobeam having a different refractive index. The refractive index determines total internal reflection and evanescent wave coupling, i.e., a higher refractive index means an easier condition for the total internal reflection and a stronger evanescent field. In Fig. S4 we present the controllability of polarization when varying the gap and shift. For example, the smallest polarization degree at the refractive index of 3.5 corresponds to the minimum value in the map of po-

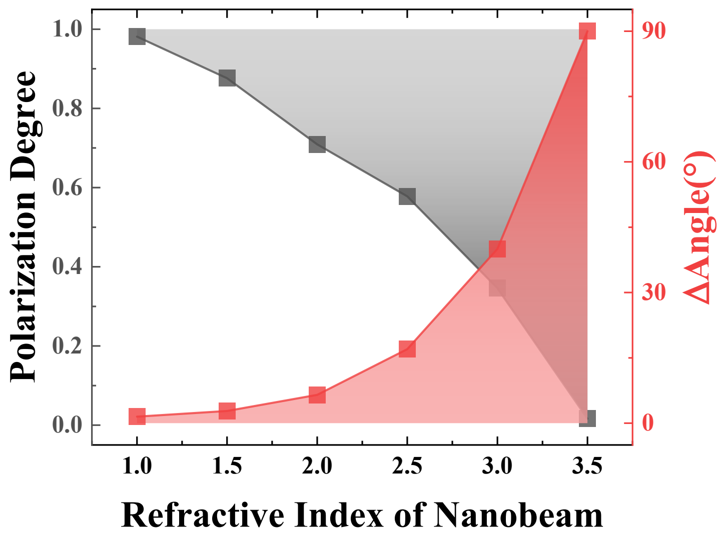

FIG. S4. Polarization controllability is calculated with different refractive index of the nanobeam. The larger refractive index means the stronger evanescent field near the nanobeam. The refractive index of 3.5 corresponds to the GaAs nanobeam. In this case the polarization degree can be tuned from 0 to 1 (circular to linear) and ΔAngle from 0 to 90 ° (y to x direction). The refractive index of 1 corresponds to the homogeneous medium. In this case the polarization is always linear and along the y direction.

larization degree in Fig. 5(b), and the ΔAngle corresponds to the maximum shift from 90° (y polarized) extracted from the map of polarization angle in Fig. 5(b). The polarization degree is defined by $|a-b|/|a+b|$, where 1 refers to linear polarization and 0 means circular polarization. The polarization angle is defined by ϕ with values ranging from 0° to 180°, in which 90° means y polarized. As shown in Fig. S4, the controllability of polarization increases with the refractive index, further supporting the conclusion that the polarization control arises from the evanescent wave coupling.

The AS mode significantly alters the symmetric properties of the field profile for the PM, and thereby, is used for the control of polarization as shown in the main text. In contrast, when the shift is zero, $s = 0$, the S mode does not change the symmetry of the field profile. As such, the S mode should exhibit linear polarization along the y direction, which is the same as that of a single cavity. This is because in the highly symmetric cases, the electric

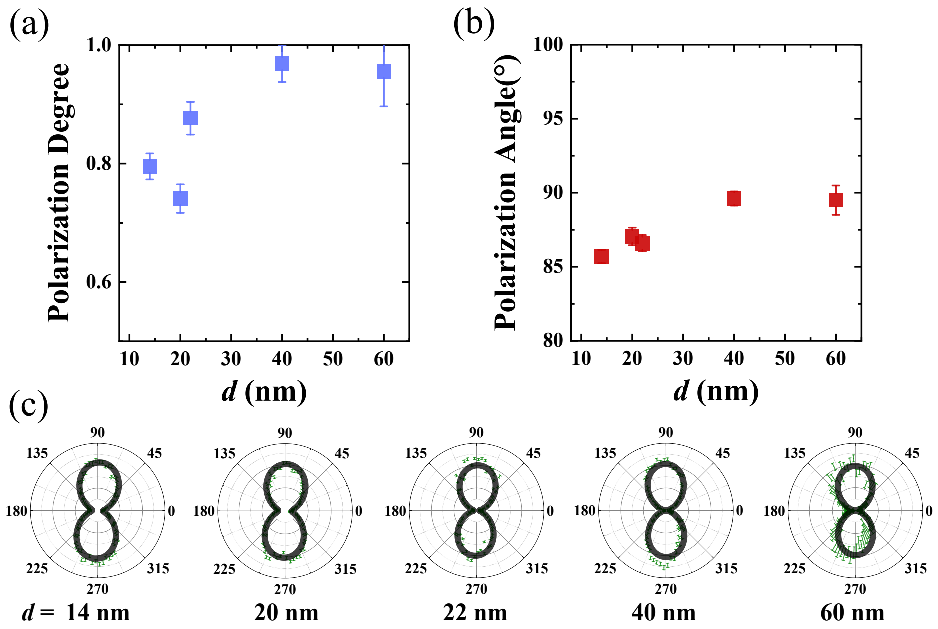

FIG. S5. Experimentally measured polarization of photons emitting from the first S mode.

fields E_θ, E_ϕ in the far-field radiation are expressed by [1]

$$E_\theta = -i\frac{e^{ikr_0}}{2\lambda r_0}\left(\eta N_\theta + L_\phi\right)$$
$$E_\phi = -i\frac{e^{ikr_0}}{2\lambda r_0}\left(-\eta N_\phi + L_\theta\right)$$

where θ, ϕ are the angles in the spherical coordinates, r_0 is the distance to the cavity center, λ is the emission wavelength, $\eta = \sqrt{\mu_0/\epsilon_0}$, and L, N are the radiation vectors for the surface electric and magnetic currents, respectively. For the out-of-plane direction $(\theta, \phi = 0)$, the electric field components are then

$$E_{far,x} = -i\frac{e^{ikr_0}}{2\lambda r_0}\left(\eta N_x + L_y\right)$$
$$E_{far,y} = -i\frac{e^{ikr_0}}{2\lambda r_0}\left(-\eta N_y + L_x\right)$$

and the radiation components are the Fourier transforms of the cavity modes as

$$N_x = -\iint H_y dxdy, N_y = \iint H_x dxdy$$
$$L_x = \iint E_y dxdy, L_y = -\iint E_x dxdy$$

in which the magnetic fields $H_{x,y}$ are zero for the TE mode. Therefore, for the symmetric E_y and anti-symmetric E_x, we can obtain a non-zero value for L_x and $E_{far,y}$ whilst the values for L_y and $E_{far,x}$ are zero, i.e., y-polarized emission.

In experiment, the wavelength (energy) shift of the S and AS modes agrees well with the theoretical calculations, as shown in Fig. 2 of the main paper, allowing us to distinguish between the S and AS modes in the experiment. We experimentally extracted the polarization properties of the S mode, including the degree shown in Fig. S5(a) and the angle in (b), from the polarization resolved intensities shown in (c). It can be seen that the S mode is nearly linear, and the variation of the polarization angle is less than 5°, fully consistent with the theoretical prediction.

We emphasize that the polarization of S mode discussed above in the context of Fig. S5 can be estimated from the features of symmetry. This is because the structure of PM is symmetric without lateral shift. In contrast, for a large shift between two nanobeams, the structure becomes asymmetric, and the electric field is no longer symmetric or anti-symmetric but asymmetric. In this case, both S and AS modes are complex, and the polarization cannot be estimated from the features of symmetry. In Fig. S6, we present the calculation results of both AS and S mode for comparison. The results of the AS mode in Fig. S6(a) are reproduced from Fig. 5 in the main paper. By comparison, in Fig. S6(b), when $s_{\text{qual}} < 100$ nm the S mode is generally y-polarized. This is consistent with the estimation from the features of symmetry and the experimental results in Fig. S5. In contrast, when $s_{\text{qual}} > 100$ nm, the polarization of the S mode also varies with the coupling, because the structure of PM becomes asymmetric. The different regions in the calculation results in Fig. S6 agree well with the features of symmetry, anti-symmetry, and asymmetry of the PM structure and cavity electric field, respectively.

We next discuss the details of polarization control with relatively small values of lateral shift. This is because the large shift means longer nanobeam, which is more difficult to fabricate in experiments. In Fig. S7, we present the polarization of the AS mode with several small values (36, 72, and 108 nm) of s_{qual}. As shown in Fig. S7(a), in the case of $s_{\text{qual}} = 108$ nm, the smallest polarization degree occurs at $d_{\text{qual}} = 252$ nm with the polarization degree of 0.18. By comparison, the $s_{\text{qual}} = 36$ nm corresponds to the smallest polarization degree at the $d_{\text{qual}} = 18$ nm with the polarization degree of 0.13. The PM with a large gap is easier to fabricate compared to one with a small gap. This means that for

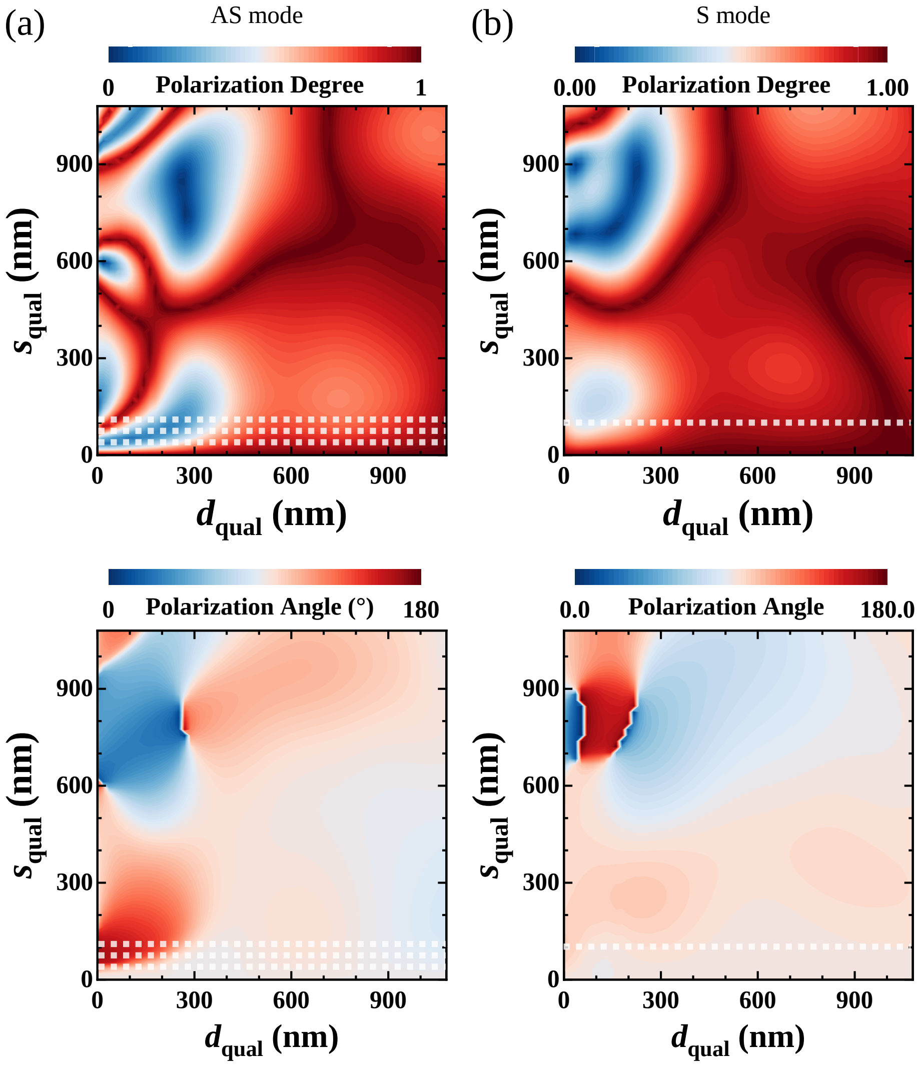

FIG. S6. Comparison of (a) AS and (b) S mode in the qualitative model in Fig. S1(a). Three dashed lines in (a) corresponds to the data in Fig. S7. The dashed line in (b) corresponds to the boundary $s_{\mathrm{qual}} = 100$ nm. When $s_{\mathrm{qual}} < 100$ nm, the structure of PM is nearly symmetric, and the S mode is generally y-polarized. In contrast, when $s_{\mathrm{qual}} > 100$ nm, the structure of PM becomes asymmetric. The cavity field of S and AS modes is no longer symmetric or anti-symmetric but asymmetric, and the polarization of S mode becomes complex.

the small $s_{\mathrm{qual}} = 36$ nm the circular polarization is difficult to achieve (needs small gap), while for the large $s_{\mathrm{qual}} = 108$ nm the circular polarization is easy to achieve (only needs large gap) but the circular polarization (degree of 0.18) is worse than in the case of $s_{\mathrm{qual}} = 36$ nm (degree of 0.13), resulting in a trade off. In Fig. S7(b), we present the result of the polarization angle. As shown, in the case of $s_{\mathrm{qual}} = 72$ nm, a nearly linear polarization (degree of 0.88) along the x direction (angle of 169°) can be achieved with the gap $d_{\mathrm{qual}} = 0$ nm. Similar polarization is achieved in the case of $s_{\mathrm{qual}} = 108$ nm when $d_{\mathrm{qual}} = 54$ nm.

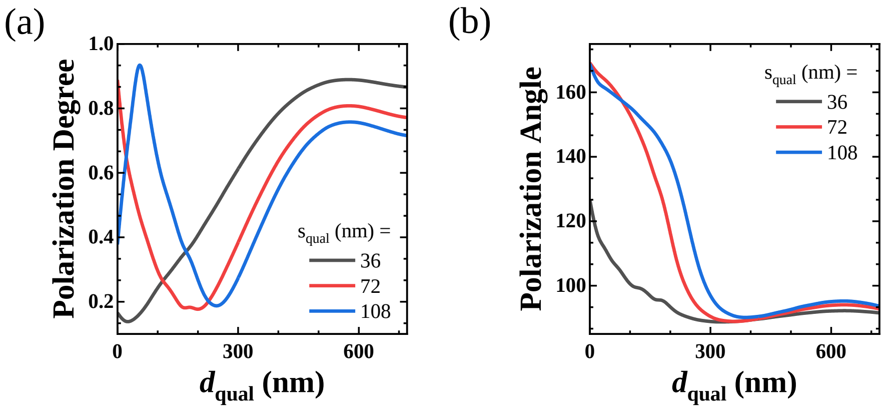

FIG. S7. The polarization data extracted from the three dashed lines in Fig. S6(a), corresponding to the cases where s_{qual} is 36, 72, and 108 nm.

In contrast, the linear polarization along the x direction is difficult to achieve for the small s_{qual} of 36 nm. These details help to improve the design and fabrication of the devices for different purpose.

[1] J. Vuckovic, M. Loncar, H. Mabuchi, and A. Scherer, Optimization of the Q factor in photonic crystal microcavities, IEEE J. Quantum Electron. **38**, 850 (2002).
